# Association of adiponectin, leptin and resistin with inflammatory markers and obesity in dementia

**DOI:** 10.1007/s10522-017-9701-0

**Published:** 2017-04-18

**Authors:** Małgorzata Bednarska-Makaruk, Ałła Graban, Anna Wiśniewska, Wanda Łojkowska, Anna Bochyńska, Magdalena Gugała-Iwaniuk, Ksenia Sławińska, Agnieszka Ługowska, Danuta Ryglewicz, Hanna Wehr

**Affiliations:** 1 Department of Genetics, Institute of Psychiatry and Neurology, Sobieskiego 9, 02-957 Warsaw, Poland; 2 First Department of Neurology, Institute of Psychiatry and Neurology, Sobieskiego 9, 02-957 Warsaw, Poland

**Keywords:** Adipokines, Dementia, Inflammatory markers, Abdominal obesity

## Abstract

The aim of the study was to determine the role of adiponectin, leptin and resistin in various types of dementia and to investigate their association with inflammatory markers, insulin resistance and abdominal obesity. In 205 patients with dementia [89 with Alzheimer’s disease (AD), 47 with vascular dementia (VaD), 69 with mixed dementia (MD)], 113 persons with mild cognitive impairment and in 107 controls serum adiponectin, leptin and resistin levels, pro-inflammatory [interleukin-6 (IL-6), C-reactive protein (hsCRP) and chitotriosidase] and anti-inflammatory (25-OH vitamin D, HDL-cholesterol and paraoxonase 1) markers, as well as glucose metabolism parameters (glucose, insulin and HOMA-IR) were determined. In all-cause dementia adiponectin and resistin levels were significantly higher as compared to the controls; leptin levels did not show differences. Higher adiponectin levels concerned AD and MD, whereas higher resistin-VaD and MD. After stratification by abdominal obesity the differences in adiponectin levels remained significant in subjects without obesity. In all-cause dementia negative correlation of adiponectin with obesity, glucose metabolism parameters, IL-6 and hsCRP and positive correlation with HDL-cholesterol were found. Positive correlation of resistin with age, IL-6, hsCRP and chitotriosidase and negative correlation with HDL-cholesterol and paraoxonase 1 were stated. We conclude that dementia of neurodegenerative origin is characterized by elevated adiponectin levels, whereas dementia with vascular changes by increase of resistin. Association with inflammatory indicators may suggest the pro-inflammatory role of resistin in the development of dementia, especially in dementia of vascular mechanism. Identification of this novel biomarker may be important in preventing dementia.

## Introduction

With aging, an increased prevalence of a clustering of metabolic abnormalities has been observed. These abnormalities including abdominal obesity, dyslipidemia, hypertension, and insulin resistance are collectively known as metabolic syndrome (MetS). MetS is regarded as a low-grade, systemic, inflammatory condition associated with an increased risk of atherosclerosis, cardiovascular disease and diabetes (Grundy et al. 2005). An increasing body of epidemiological evidence suggested that MetS and its individual components could be linked to a risk of developing age-related cognitive decline, mild cognitive impairment, vascular dementia, and Alzheimer’s disease. Recently the presence of a “metabolic-cognitive syndrome”, i.e. a MetS plus cognitive impairment of degenerative or vascular origin disease was proposed (Frisardi et al. 2010; Parimisetty et al. 2016). Accumulating evidence suggests that obesity (particularly central or abdominal obesity) contributes to chronic inflammation, leading to the development of insulin resistance and the metabolic syndrome (Wellen and Hotamisligil 2003).

Individuals with obesity exhibit a dysregulation of adipokines, which are highly bioactive substances secreted by adipocytes and immune cells. An increasing number of adipokines is described in the literature (Deng and Scherer 2010). Adipokines exert pleiotropic effects on different tissues and regulate numerous important physiological functions such as appetite, energy expenditure, insulin sensitivity and secretion, fat distribution, lipid and glucose metabolism, endothelial function, blood pressure, hemostasis, neuroendocrine functions and also immunity (Deng and Scherer 2010; Blüher and Mantzoros 2015).

It is acknowledged now that adipokines have more widespread influence and functionality in the brain than previously thought. The association between adipokines and clinical dementia or cognitive impairment is largely unexplored, despite published epidemiological data supporting associations between obesity and various types of dementia and Alzheimer’s disease. (Parimisetty et al. 2016). Several case–control and large population based prospective studies have shown an association of lower circulating leptin levels with AD or general cognitive decline (Lieb et al. 2009; Baranowska-Bik et al. 2015), but some clinical studies found no differences in AD and controls (Theodoropoulou et al. 2012; Warren et al. 2012; Teunissen et al. 2015). In case of adiponectin the epidemiological link with AD risk is less clear. Some case–control and large follow-up studies have shown an association of higher circulating or cerebrospinal fluid level of adiponectin with increased risk of AD (Une et al. 2011; van Himbergen et al. 2012; Khemka et al. 2014), but other authors found no difference in adiponectin level between patients with AD, VaD, MCI and healthy controls (Warren et al. 2012; Bigalke et al. 2011; Dukic et al. 2016). In contrast, in the study of Teixeira et al. (2013) decreased adiponectin serum concentrations are associated with MCI and AD.

Experimental studies in cell lines and AD transgenic mice indicated that leptin can affect APP-Aβ trafficking, Aβ accumulation and clearance, β-secretase expression and phosphorylation of tau protein (Chacrabarti et al. 2015). Contrary to leptin, the role of adiponectin in the brain has not yet been well elucidated. Exogenous adiponectin was reported to be protective against Aβ-induced neurotoxicity under oxidative stress conditions in a cell model (Chan et al. 2012). Adiponectin could modulate brain metabolism and sensitivity of insulin regulating memory and cognitive dysfunction (Song and Lee 2013).

Resistin, although classified as an adipokine, in humans is expressed in macrophages and plays important roles in inflammation throughout the body. Its serum level is increased in cardiovascular disease (Karbowska et al. 2009; Reilly et al. 2005), other inflammation-related diseases (Pang and Lee 2006) and in diabetes (Rajala et al. 2004), however, studies relating human resistin to insulin resistance, or diabetes have shown conflicting results.

The impact of resistin on the physiology and the pathophysiology of the central nervous system remains poorly defined. The potential protective effects of resistin against Aβ neurotoxicity in mouse cells which overproduced Aβ was stated by Liu et al. (2013). The results of the two clinical studies are inconclusive. A recent study of Mirabell et al. (2013) suggested that resistin was not related to cognitive function performance, whereas according to another study AD was associated with increased serum resistin levels (Kizilarslanoglu et al. 2015).

Epidemiological and clinical studies have suggested that chronic inflammation may have an important role in dementia (Solfrizzi et al. 2006; Tan et al. 2007). Several pathways through which inflammation can drive AD pathogenesis have been identified (Lee et al. 2009). For example, increased levels of pro-inflammatory cytokines can stimulate amyloid precursor protein (APP) processing to generate more Aβ, which not only led to neurodegeneration, but also acts on microglia and astrocytes to further increase inflammation (Blasko et al. 2000). Recently vitamin D, which was stated to be a neuro-immunomodulator, was suggested to play a role in the pathogenesis of various brain diseases (Fernandes de Abreu et al. 2009). Accumulating evidence indicates that anti-oxidant and anti-inflammatory properties of high density lipoproteins (HDL) may play significant role in neuroprotection and maintaining cognitive function during aging (Hottman et al. 2014). It was shown that paraoxonase 1 (PON1), an enzyme carried by HDL, mainly contribute to antioxidant and anti-inflammatory properties of the particle (Aharoni et al. 2013). The enzyme chitotriosidase, a nonspecific marker of macrophage activation, is recently considered to be a novel marker of inflammation (Kanneganti et al. 2012).

The aim of the present study was to investigate the serum levels of selected adipokines (adiponectin, leptin and resistin) in various types of dementia and mild cognitive impairment in relation to abdominal obesity—the most important feature of metabolic syndrome. We also explored the association of adipokines with some pro- (interleukin-6, C-reactive protein, chitotriosidase) and anti-inflammatory markers (HDL-cholesterol, paraoxonase 1, 25-OH vitamin D) and insulin resistance indices (glucose, insulin, HOMA-IR index).

## Materials and methods

### Subjects

The investigated group consisted of 425 elderly subjects. The whole group with dementia included 205 consecutive patients (75 males and 130 females) aged 74.5 ± 8.23 years, admitted to the First Department of Neurology, Institute of Psychiatry and Neurology in Warsaw between June 2012 and December 2015. There were 89 patients (mean age 72.8 ± 8.13 years) diagnosed as probable Alzheimer’s disease, 47 (mean age 74.9 ± 9.00 years) as dementia of vascular origin and 69 (mean age 76.5 ± 7.39 years) as mixed dementia. 113 persons aged 70.5 ± 8.89 years were recognized as having mild cognitive impairment. 107 persons aged 71.3 ± 7.95 years, without any cognitive deficits and in good general health, matched to dementia patients for age, constituted the control group. The control subjects were mainly recruited from healthy volunteers, healthy spouses of demented patients as well as from patients from the outpatient clinic, in whom dementia or MCI were excluded basing on neuropsychological test.

Dementia was recognized according to ICD-10 and DSM-IV criteria. Mini Mental State Examination (MMSE) and a Clock Drawing Test were used as screening tests for existing dementia in all participants. All patients underwent a general physical and neurological evaluation, neuroimaging examinations (computer tomography-CT or magnetic resonance-MRI) and comprehensive neuropsychological assessment. The type of dementia was diagnosed based on the NINCDS-ADRDA scale for AD and NINDS-AIREN for VaD. MD was defined as coexisting cerebrovascular disease (CVD) in AD patients, defined as significant radiological evidence of CVD in CT or MRI. For MCI diagnosis, the Petersen criteria were used (Petersen and Negash 2008).

All participants were Polish and were white. Most of the subjects were living in urban area (87.3% dementia patients, 95.6% MCI subjects and 95.3% controls), mainly in Warsaw and the suburbs. Majority of investigated individuals were home-dwelling persons and only four patients with dementia were nursing-home residents. Over 90% participants were retired and in most of them physical activity is limited to daily activities.

In all study participants demographic and lifestyle data, anthropometric measures and clinical anamnesis were obtained by physicians and nursing staff. Past and current disease history addressed major age-related disorders i.e. cardiovascular disease (coronary artery disease including myocardial infarction, stroke or TIA, peripheral artery disease), hypertension, diabetes, hyperlipidemia cancer and depression.

In all subjects current use of medications was recorded including: lipid-lowering (statins and/or fibrates), anti-diabetic (sulfonylureas and/or biguanides and/or insulin), anti-hypertensive (ACE-inhibitors/angiotensin II receptor blockers and/or beta-blockers and/or calcium channel blockers and/or diuretics) drugs and drugs used in dementia treatment (cholinesterase inhibitors or memantine).

Subjects with evidence of malignant disease, myocardial infarction or stroke up to six months before recruitment, hypothyroidism, alcohol abuse, autoimmune disorders, symptoms of acute infections, therapy with steroids, severe depression and other mood disorders were excluded from the study.

The study was approved by the Ethics Committee of the Institute of Psychiatry and Neurology. Subjects gave their informed consent either directly or received it from their guardians.

### Methods

The blood samples for biochemical and molecular analyses were obtained after an overnight fast. Serum was isolated and stored at −70°C until analysis. Genomic DNA was extracted from peripheral blood leukocytes by phenol/chloroform extraction and ethanol precipitation (Maniatis et al. 1982).

Body weight and height were measured in the morning with the subject wearing light clothing and without shoes. Body mass index (BMI) was calculated as body weight (in kg) divided by height (in meters) square. Waist circumference (cm) was measured while the subjects were standing after normal expiration.

Serum adiponectin, leptin and resistin levels were measured by solid-phase sandwich enzyme immunoassay technique (R&D Systems Inc., USA). Serum IL-6 (R&D Systems Inc., USA) and CRP (DRG Instruments GmbH, Germany) were measured by high sensitivity solid-phase ELISA assays. Serum insulin was measured by a solid phase enzyme-linked immunosorbent assay (DRG Instruments GmbH, Germany), based on the sandwich principle. Serum 25-OH Vitamin D was measured by a solid phase enzyme-linked immunosorbent assay (DRG Instruments GmbH, Germany), based on the principle of competitive binding. All ELISA kits were commercially available, and assays were carried out according to manufacturer’s instructions.

Leptin to adiponectin ratio (L/A) (which was shown to be a better biomarker for MetS diagnosis criteria than leptin and adiponectin separately) was calculated.

HOMA-IR-homeostatic model assessment index (which was shown to correlate well with insulin sensitivity measured by the euglycemic clamp method) was calculated as follows: fasting glucose (mmol/L) x fasting insulin (mU/L)/22.5 (Muniyappa et al. 2008).

Glucose was determined using enzymatic method after an overnight fast and after 75 g glucose load (oral glucose tolerance test-OGTT). OGTT was not performed in subjects with previously diagnosed diabetes. Diabetes mellitus (DM) was defined according to American Diabetes Association guidelines (2010) by a recorded fasting glucose ≥7.0 mmol/L (126 mg/dL) or post-load/casual blood glucose level of at least 200 mg/dL ≥ 11.1 mmol/L (200 mg/dL) or a previous diagnosis of diabetes mellitus or the use of an oral hypoglycemic agent or insulin.

Serum triglycerides and cholesterol were determined in the fresh serum by enzymatic methods. HDL cholesterol was determined after removing apolipoprotein B containing lipoproteins by precipitation.

Metabolic syndrome was defined by IDF (International Diabetes Federation) and AHA/NHLBI (American Heart Association/National Heart, Lung, and Blood Institute) joint scientific statement criteria (Alberti et al. 2009). The following abnormalities were taken into account: (1) abdominal obesity with waist circumference ≥102 cm in men and ≥88 cm in women; (2) elevated triglyceride (TG): ≥150 mg/dL (1.7 mmol/L) or drug treatment for elevated TG; (3) reduced HDL cholesterol (HDL-C): <40 mg/dL (1.03 mmol/L) in men, <50 mg/dL (1.3 mmol/L) in women or drug treatment for reduced HDL; (4) elevated blood pressure (BP): systolic BP ≥ 130 mm Hg or diastolic BP ≥ 85 mm Hg or drug treatment for hypertension; (5) elevated fasting glucose ≥100 mg/dL or hypoglycemic treatment. The presence of three out of five abnormal findings constituted a diagnosis of MetS.

Paraoxonase 1 activity was determined spectrophotometrically based on the Kitchen et al. method (1973) using phenyloacetate as substrate. One unit of activity was 1 μmol of phenol liberated per minute per 1 mL of serum.

Serum activity of chitotriosidase was measured by a spectrofluorometric method according to Hollak et al. (1994) using the synthetic substrate 4-methylumbelliferylb-N–N′-N″-triacetylchitotrioside (Sigma Chemical Co, St. Louis, MO). Fluorometric measurements were made at excitation λ = 365 nm and emission λ = 445 nm (Hitachi, Japan).

Duplication of 24-bp (dup24 bp) in exon 10 of the chitotriosidase gene (*CHIT1*) (rs3831317) was identified by duplication mutation analysis using PCR according to Boot et al. (1998). DNA fragments amplified from the normal and mutant alleles, were identified after electrophoresis in 3% agarose gel. The identification of chitotriosidase variant form is indispensable because it is strongly influencing the activity of the enzyme-homozygotes of the mutant form display extremely low activity (Boot et al. 1998).

APOE ε2/ε3/ε4 polymorphism (rs7412 and rs429358) was investigated using the PCR–Restriction Fragment Length Polymorphism (PCR–RFLP) method according to Hixon and Vernier (1990), consisting of amplification of the *APOE* gene fragment, its enzymatic cleavage by Hin6I restriction enzyme (Fermentas, Lithuania) and identification of DNA fragments after electrophoresis on 10% polyacrylamide gel.

### Statistical analysis

Statistical analysis was performed using Statistica version 12 (StatSoft). Shapiro and Wilk test was carried out to ascertain the normality of the distribution of continuous variables. Normally-distributed variables were presented as mean ± SD. Not-normally-distributed variables were presented as geometric means with interquartile ranges (IQR) and were logarithmically transformed to approximate a normal distribution before statistical analysis, but the results were expressed as crude data. Student *t* test or One-way Analysis of Variance (ANOVA) followed by Dunnett’s post hoc test was carried out to assess differences for continuous variables among subjects with various types of dementia, MCI and controls. The mean levels of adipokines and glucose metabolism parameters were also adjusted for age, sex and BMI and the anti-hypertensive, anti-diabetic and lipid-lowering treatment using the covariance analysis (ANCOVA) with above mentioned variables as covariates. The mean levels of hsIL-6 and hsCRP were adjusted for age, current smoking status and the anti-hypertensive, anti-diabetic and lipid-lowering treatment and mean level of 25-OH vitamin D was adjusted for age, sex, season and geographical region (ANCOVA). Factorial Two-way ANOVA was used to evaluate effects of two categorical variables (i.e. the presence of abdominal obesity and dementia diagnosis) on one continuous variable (i.e. each adipokine level). Statistical significance of the differences in the frequencies of qualitative variables was evaluated using Pearson’s *χ* ^2^ test. Pearson correlation coefficients were calculated to describe associations of plasma adipokines with metabolic and inflammatory indices as well as with MMSE score among dementia and control subjects (univariate analysis). The associations between various types of dementia and adipokines were identified using univariate and multiple logistic regression analysis with age, sex, level of education and the presence of the *APOE* ε4 allele and the use of anti-hypertensive, anti-diabetic and lipid-lowering therapy as confounding factors and presented as odds ratios (OR) with 95% confidence intervals (CI). Multivariate forward stepwise regression analysis was performed to assess the independent effects of the different factors on serum adiponectin, leptin and resistin levels in whole investigated group. The following variables (chosen on the basis of previous univariate analyses) were included in the model: for adiponectin analysis BMI, HDL cholesterol, HOMA-IR, hsIL-6, for leptin analysis BMI, HDL cholesterol (HDL-C), HOMA-IR, hsCRP and for resistin analysis HDL cholesterol, IL-6, 25(OH) vitamin D, creatinine, serum chitotriosidase, current smoking. Moreover in all analyses age, sex, anti-hypertensive, anti-diabetic and lipid-lowering therapy and the type of dementia were included.

p values lower than 0.05 were considered as statistically significant.

## Results

### Clinical and biochemical characteristic of the study groups

Demographic and clinical characteristic of the study groups are presented in Table 1. Patients with all-cause dementia and particularly with MD were older than controls. There were no significant differences in gender distribution among investigated groups. All patients with dementia (AD, MD and VaD) were less educated, had worse cognitive performance and showed a higher frequency of the *APOE* ε4 allele when compared to the control group. Patients with VaD had significant higher prevalence of hypertension and cardiovascular disease, whereas in AD the prevalence of cardiovascular disease was significantly lower as compared to controls. Moreover the percentage of VaD subjects on anti-hypertensive therapy was also significantly higher than controls. There were no significant differences in proportion of current smokers as well as in mean body mass index (BMI) among investigated groups. The prevalence of abdominal obesity was borderline higher in MCI (63.7%) as compared to controls (52.3%).

**Table 1:** Demographic and clinical characteristics of the study subjects

Variables	Dementia	p^a^	AD	MD	VaD	MCI	Controls	p^b^
	n = 205		n = 89	n = 69	n = 47	n = 113	n = 107	
Age (years)	74.5 ± 8.23	0.0009	72.8 ± 8.13	76.5 ± 7.39***	74.9 ± 9.00	70.5 ± 8.89	71.3 ± 7.95	0.00001
Sex (% men)	36.6	0.852	25.8	46.4	42.6	35.4	35.5	0.095
Education ≥ 12 years (%)	66.5	0.00002	76.1*	58.0***	60.9***	83.0	88.8	<0.00001
MMSE (score)	18.1 ± 5.99	<0.00001	17.3 ± 6.17***	17.3 ± 5.76***	21.1 ± 5.12***	27.2 ± 1.96	29.2 ± 1.18	<0.00001
APOE ε4 (%)	46.5	<0.00001	56.5***	43.5***	32.6#	28.6	19.1	<0.00001
Vascular risk factors								
Current smokers (%)	3.9	0.811	3.4	4.4	4.3	9.7	4.3	0.222
Hypertension (%)	58.5	0.795	47.2	58.0	80.9**	67.3	57.0	0.001
BMI (kg/m^2^)	26.3 ± 4.56	0.733	25.7 ± 4.49	26.4 ± 4.97	27.1 ± 3.98	27.4 ± 4.33	26.4 ± 3.84	0.056
Abdominal obesity (%)	48.8	0.551	44.9	43.5	63.8	63.7#	52.3	0.016
Cardiovascular disease (%)	33.7	0.311	12.4**	34.8	72.3***	35.4	28.0	<0.00001
Diabetes mellitus (%)	27.3	0.014	20.2	27.6*	40.4***	27.4*	15.0	0.009
MetS (%)	31.7	0.990	24.7	29.0	48.9*	39.8	31.8	0.023
Pharmacological therapy								
Anti-hypertensive therapy (%)	55.1	0.639	42.7	55.1	78.7**	66.4*	52.3	0.0002
Lipid-lowering therapy (%)	40.5	0.833	39.3	34.8	51.1	46.0	39,3	0.356
Anti-diabetic therapy (%)	13.2	0.620	10.1	10.1	23.4#	22.1	11.2	0.024
Dementia therapy (%)	25.9	<0.00001	38.2	18.8	12.8	0	0	<0.00001

Values are presented as mean ± standard deviations or percentages

*AD* Alzheimer’s disease, *APOE* apolipoprotein E, *MCI* mild cognitive impairment, *MD* mixed dementia, *MetS* metabolic syndrome, *MMSE* mini mental state examination, *VaD* vascular dementia

* p < 0.05, ** p < 0.01, *** p < 0.001, # borderline significant (0.1 > p > 0.05) versus Controls (ANOVA Dunnet post hoc test or *χ* ^2^ test)

^a^Student’s *t* test or *χ* ^2^ test

^b^ANOVA or *χ* ^2^ test

The prevalence of diabetes mellitus was significantly higher in whole group of dementia than in the control group (27.3 vs. 15.0%, p = 0.014), particularly concerning dementia with vascular changes (VaD and MD) and also MCI. Metabolic syndrome was significantly more frequent only in patients with VaD (48.9 vs. 31.8% in controls, p = 0.023).

Table 2 shows the inflammatory and glucose metabolism markers in various types of dementia, MCI and controls. The whole dementia group in comparison with controls was characterized by statistically significant higher levels of pro-inflammatory IL-6 (p = 0.016). Significantly higher levels of IL-6 concerned dementia with vascular component (VaD and MD). The differences remained significant after adjusting the means for age and smoking using covariance analysis (ANCOVA). There was a tendency towards higher hsCRP levels in mixed dementia group. No significant differences in serum chitotriosidase activity between the investigated groups were found.

**Table 2:** Inflammatory and glucose metabolism parameters in various types of dementia, MCI and controls

Variables	Dementia	p^a^	AD	MD	VaD	MCI	Controls	p^b^
	n = 205		n = 89	n = 69	n = 47	n = 113	n = 107	
Inflammatory markers								
IL-6 (pg/mL)	1.99 [1.15–3.05] (1.88)	0.016 (0.258)^c^	1.57 [0.79–2.54] (1.60)	2.39 [1.22–3.96]** (2.17)**	2.37 [1.49–3.77]* (2.26)*	1.61 [0.96–2.38] (1.73)	1.58 [0.95–2.20] (1.68)^h^ (1.68)^i^	0.0005 (0.033)^c^
hsCRP (mg/L)	1.52 [0.50–3.89] (1.43)	0.572 (0.845)^c^	0.95 [0.36–2.44] (0.96)	2.22 [0.63–7.41]# (2.06)#	2.11 [0.79–4.61] (2.04)	1.43 [0.68–3.37] (1.50)	1.39 [0.78–2.92] (1.48)^h^ (1.46)^i^	0.001 (0.004)^c^
25-OH Vitamin D (ng/mL)	18.74 [14.13–24.72] (18.85)	0.718 (0.884)^d^	20.08 [15.11–26.05] (19.73)	17.41 [13.97–23.31] (17.63)	18.29 [13.43–24.53] (18.63)	22.00 [16.18–29.81]# (21.93)*	19.11 [15.97–26.49] (20.00)^h^ (18.93)^i^	0.008 (0.015)^d^
HDL-C (mg/dL)	61.5 [51.2–75.1] (62.0)	0.602 (0.324)^e^	66.6 [56.2–78.6]# (63.8)#	58.0 [46.3–70.9] (58.3)	57.9 [49.2–71.1] (60.3)	60.9 [52.0–74.5] (61.6)	60.5 [52.0–73.8] (60.1)^h^ (59.5)^i^	0.030 (0.302)^e^
PON1 (U/mL)	158.1 [139–190]	0.134	169.4 [149–199]	147.7 [130–180]*	153.5 [130–190]	162.3 [138–194]	166.4 [144–207]	0.016
Chitotriosidasex (nmol/mL/H)^f^	72.6 [50–118]	0.891	68.8 [44–110]	80.6 [56–124]	69.1 [49–122]	60.1 [35–100.5]	71.9 [46–107]	0.054
Glucose metabolism parameters								
Glucose 0H (mg/dL)	103.0 [91.2–109.0] (102.2)	0.020 (0.076)^g^	103.4 [92.6–108.5] (105.8)	101.5 [91.0–106.1] (102.2)	104.4 [91.7–112.4] (100.9)	104.4 [92.3–110.6]# (102.8)	97.9 [89.7–103.5] (98.7)^h^ (99.9)^i^	0.117 (0.135)^g^
Glucose 2H (mg/dL)	138.6 [109.2–177.8] (136.0)	0.000001 (0.0002)^g^	132.7 [105.9–167.8]** (134.4)**	144.5 [110.7–182.5]*** (143.0)***	142.5 [116.9–193.8]** (136.3)**	120.3 [95.4–150.9] (122.0)	113.7 [98.3–131.8] (115.9)^h^ (116.8)^i^	0.00003 (0.0007)^g^
Insulin (μIU/mL)	9.01 [5.84–13.60] (9.24)	0.654 (0.231)^g^	9.31 [6.42–13.72] (9.82)	9.06 [5.52–15.03] (9.21)	8.40 [5.50–12.31] (8.25)	9.12 [5.62–15.05] (8.64)	8.72 [5.62–12.76] (8.51)^h^ (8.74)^i^	0.903 (0.493)^g^
HOMA-IR	2.29 [1.46–3.52] (2.33)	0.309 (0.123)^g^	2.38 [1.64–3.56] (2.57)	2.27 [1.35–3.76] (2.32)	2.17 [1.17–3.34] (2.05)	2.35 [1.43–4.12] (2.19)	2.11 [1.38–3.17] (2.07)^h^ (2.16)^i^	0.756 (0.291)^g^

Data are logarithmically transformed in statistical analysis and presented as geometric means and interquartile range from first to third quartile

Values in parentheses are means adjusted by various covariates (ANCOVA)

*AD* Alzheimer’s disease, *CRP* C-reactive protein, *HDL-C* high density lipoprotein cholesterol, *HOMA-IR* homeostatic model assessment index, *IL-6* interleukin 6, *MCI* mild cognitive impairment, *MD* mixed dementia, *PON1* paraoxonase 1, *VaD* vascular dementia

* p < 0.05, ** p < 0.01, *** p < 0.001, # borderline significant (0.1 > p>0.05) versus Controls (ANOVA Dunnet post hoc test or *χ* ^2^ test)

^a^Student’s *t* test

^b^ANOVA

^c^Adjusted for age and smoking

^d^Adjusted for age, sex, BMI, season, region (town or country)

^e^Adjusted for age, sex, anti-diabetic, anti-hypertensive and lipid-lowering, therapy

^f^Homozygotes of *CHIT*124-bp dup excluded (n = 18: AD n = 4; MD n = 3; VaD n = 1; MCI n = 5; controls n = 6)

^g^Adjusted for age, sex, BMI, anti-diabetic, anti-hypertensive and lipid-lowering, therapy

^h^Adjusted values in controls versus all-cause dementia

^i^Adjusted values in controls versus various types of dementia and MCI

There were no significant differences in the levels of anti-inflammatory markers: 25-OH vitamin D, HDL-cholesterol between whole dementia and various types of dementia and the control group. A tendency to higher serum 25-OH vitamin D levels was observed in MCI as compared to controls, the difference reach statistical significance after adjustment the means for age, sex, season, region and BMI. PON1 activity was significantly lower in MD as compared to controls.

Blood glucose both the fasting (p = 0.020) and particularly 2-h post load concentrations (p = 0.000001) were significantly higher in all patients with dementia, but the levels of insulin and insulin resistance index (HOMA-IR) showed no differences as compared to the control group. A significantly higher 2-h post load glucose level was observed in all types of dementia as compared to the control group. There were no statistically significant differences in the levels of fasting glucose, insulin and HOMA-IR between the various types of dementia, MCI and the control group.

### Serum adiponectin, leptin and resistin in dementia and healthy controls

The serum levels of investigated adipokines in all studied groups are shown in Table 3. In the whole dementia group the mean levels of adiponectin (p = 0.002) and resistin (p = 0.00009) were significantly higher and leptin/adiponectin (L/A) ratio (p = 0.015) was significantly lower as compared to the control group. In comparison with the controls, significantly higher concentrations of adiponectin were stated in dementia of neurodegenerative origin (AD and MD), and significantly higher levels of resistin were observed in dementia with vascular changes (VaD and MD). The differences remained significant after adjusting the means age, sex, BMI and anti-hypertensive, anti-diabetic and lipid-lowering therapy using covariance analysis (ANCOVA) with above mentioned variables as covariates. There were no significant differences in leptin levels between whole dementia, different types of dementia and MCI as compared to the control group. The L/A ratio was borderline significantly lower in AD.

**Table 3:** Adiponectin, leptin, leptin/adiponectin ratio and resistin and in various types of dementia, MCI and controls

Adipokines	Dementia	p^a^	AD	MD	VaD	MCI	Controls	p^b^
	n = 205		n = 89	n = 69	n = 47	n = 113	n = 107	
Adiponectin (μg/mL)	10.20 [6.79–13.80] (9.44)	0.002 (0.002)	11.44 [7.28–15.71]** (9.19)***	10.30 [6.99–13.61]** (9.63)**	8.39 [4.85–12.27] (8.39)	8.12 [4.81–12.01] (8.69)	8.23 [4.87–11.63] (7.66)^c^ (7.45)^d^	0.001 (0.019)
Leptin (ng/mL)	8.66 [3.81–17.36] (7.99)	0.357 (0.365)	8.13 [4.15–15.38] (8.18)	8.66 [3.81–17.40] (8.98)	10.08 [3.36–19.62] (7.46)	10.18 [5.01–17.32] (8.36)	9.96 [5.48–15.36] (8.58)^c^ (8.88)^d^	0.551 (0.498)
L/A ratio	0.83 [0.31–2.40] (0.85)	0.015 (0.012)	0.72 [0.31–2.01]# (0.89)	0.93 [0.32–2.12] (0.93)	1.05 [0.31–3.89] (0.89)	1.33 [0.52–3.15] (0.96)	1.31 [0.58–2.56] (1.12)^c^ (1.19)^d^	0.032 (0.149)
Resistin (ng/mL)	9.22 [7.39–11.96] (9.37)	0.00009 (0.005)	8.32 [6.95–10.94] (9.15)	9.87 [7.76–12.53]*** (9.78)***	9.62 [7.52–14.02]** (9.64)**	8.29 [6.25–10.75] (8.56)	8.43 [6.50–10.04] (8.29)^c^ (8.33)^d^	0.0001 (0.018)

Data are logarithmically transformed in statistical analysis and presented as geometric means and interquartile range from first to third quartile

Values in parentheses are adjusted on age, sex, BMI, anti-diabetic, antihypertensive and lipid-lowering treatment (ANCOVA)

*AD* Alzheimer’s disease, *L/A ratio* leptin/adiponectin ratio, *MCI* mild cognitive impairment, *MD* mixed dementia, *VaD* vascular dementia

* p < 0.05, ** p < 0.01, *** p < 0.001, # borderline significant (0.1 > p > 0.05) versus Controls (ANOVA Dunnet post hoc test)

^a^Student’s *t* test

^b^ANOVA

^c^Adjusted values in controls versus all-cause dementia

^d^Adjusted values in controls versus various types of dementia and MCI

To test whether plasma levels of adipokines (leptin, adiponectin and resistin) are independently associated with the presence of various types of dementia, we performed a univariate and multivariate logistic regression analysis including known dementia risk factors such as age, gender, level of education and APOE ε4 allele as confounding variables (Table 4). All subjects were stratified according to tertiles of adiponectin, leptin, L/A ratio and resistin. Both the crude odds ratios (ORs) and ORs after adjusting for age, sex, years of education and APOE ε4 allele indicate that higher adiponectin levels were significantly associated with the increased risk of all-cause dementia, AD and MD, but not with VaD and MCI. The all-cause dementia, AD and MD risk were over 3 times greater in third tertile as compared with the first tertile of serum adiponectin levels adjusted. The risk of all-cause dementia and AD decreased progressively with increased L/A ratio from the first to the third tertile (in all-cause dementia adjusted OR = 0.45 and in AD OR = 0.35). In the case of resistin crude ORs showed significant association of increased levels of resistin with higher risk of all-cause dementia (OR = 2.50), MD (OR = 4.05) and VaD (OR = 3.02). However these associations were no longer statistically significant after controlling for the other risk factors. Logistic regression analysis show no association of leptin levels with risk of dementia.

**Table 4:** Odds ratios and 95% confidence intervals for adiponectin, leptin, leptin/adiponectin ratio and resistin with dementia and MCI

	Dementia		AD		MD		VaD		MCI	
	n = 205		n = 89		n = 69		n = 47		n = 113	
	A	B	A	B	A	B	A	B	A	B
	OR [95%CI]	OR [95%CI]	OR [95%CI]	OR [95%CI]	OR [95%CI]	OR [95%CI]	OR [95%CI]	OR [95%CI]	OR [95%CI]	OR [95%CI]
Adiponectin (μg/mL)										
1st Tertile (≤6.83)	1	1	1	1	1	1	1	1	1	1
2nd Tertile (6.84–11.70)	1.67 [1.25–2.25]	1.77 [1.26–2.48]	1.86 [1.30–2.67]	1.78 [1.17–2.72]	1.79 [1.21–2.64]	1.93 [1.17–3.20]	1.25 [0.81–1.92]	1.24 [0.74–2.07]	1.15 [0.82–1.60]	1.17 [0.80–1.70]
3rd Tertile (>11.70)	2.81 [1.55–5.07]	3.12 [1.60–6.17]	3.47 [1.69–7.11]	3.17 [1.36–7.41]	3.19 [1.47–6.95]	3.73 [1.37–10.22]	1.56 [0.66–3.70]	1.53 [0.55–4.28]	1.32 [0.68–2.56]	1.36 [0.64–2.89]
p	0.0006	0.001	0.0006	0.007	0.003	0.010	0.304	0.411	0.410	0.425
Leptin (ng/mL)										
1st Tertile (≤5.89)	1	1	1	1	1	1	1	1	1	1
2nd Tertile (5.90–13.79)	0.90 [0.67–1.20]	0.78 [0.56–1.11]	0.87 [0.61–1.24]	0.68 [0.44–1.05]	0.90 [0.61–1.32]	0.99 [0.61–1.63]	0.92 [0.60–1.42]	0.92 [0.54–1.56]	1.64 [0.83–1.63]	1.22 [0.83–1.78]
3rd Tertile (>13.79)	0.80 [0.45–1.43]	0.61 [0.31–1.23]	0.76 [0.37–1.55]	0.46 [0.19–1.10]	0.81 [0.37–1.74]	0.98 [0.37–2.65]	0.85 [0.35–2.03]	0.85 [0.30–2.43]	1.36 [0.70–2.64]	1.48 [0.69–3.18]
p	0.452	0.165	0.442	0.079	0.583	0.976	0.709	0.756	0.369	0.310
L/A ratio										
1st Tertile (≤0.579)	1	1	1	1	1	1	1	1	1	1
2nd Tertile (0.580–1.989)	0.74 [0.56–0.99]	0.67 [0.49–0.93]	0.67 [0.47–0.96]	0.59 [0.39–0.90]	0.70 [0.47–1.03]	0.74 [0.47–1.18]	0.93 [0.60–1.42]	0.98 [0.61–1.57]	1.006 [0.73–1.38]	1.001 [0.75–1.34]
3rd Tertile (>1.989)	0.56 [0.31–0.99]	0.45 [0.24–0.87]	0.45 [0.22–0.93]	0.35 [0.15–0.80]	0.49 [0.22–1.06]	0.56 [0.22–1.40]	0.86 [0.36–2.01]	0.95 [0.37–2.46]	1.013 [0.54–1.90]	1.004 [0.56–1.81]
p	0.045	0.017	0.029	0.013	0.069	0.209	0.720	0.923	0.969	0.990
Resistin (ng/mL)										
1st Tertile (≤7.43)	1	1	1	1	1	1	1	1	1	1
2nd Tertile (7.44–10.07)	1.58 [1.17–2.13]	1.35 [0.97–1.88]	1.28 [0.90–1.82]	1.22 [0.81–1.48]	2.01 [1.34–3.01]	1.53 [0.94–2.49]	1.74 [1.11–2.71]	1.44 [0.86–2.41]	1.08 [0.78–1.51]	1.13 [0.81–1.60]
3rd Tertile (>10.07)	2.50 [1.38–4.52]	1.82 [0.93–3.55]	1.63 [0.81–3.31]	1.48 [0.66–3.33]	4.05 [1.81–9.08]	2.35 [0.89–6.18]	3.02 [1.24–7.36]	2.08 [0.75–5.79]	1.18 [0.61–2.28]	1.29 [0.65–2.55]
p	0.002	0.078	0.171	0.341	0.0006	0.082	0.014	0.158	0.629	0.466

*AD* Alzheimer’s disease, *MCI* mild cognitive impairment, *MD* mixed dementia, *VaD* vascular dementia, *A* not adjusted values, *B* values adjusted for age, sex, *APOE* ε4 allele, education

### Influence of abdominal obesity on adipokine levels in dementia and healthy controls

**Fig. 1:**
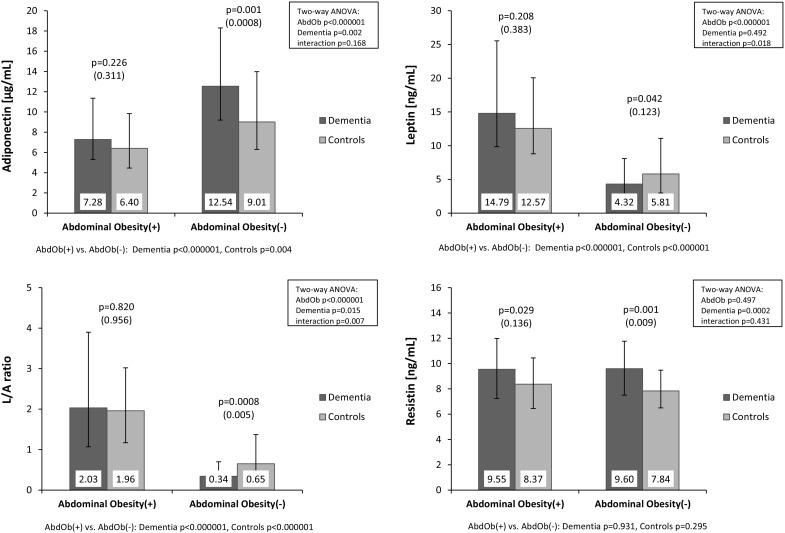
Serum adiponectin, leptin, resistin levels and leptin/adiponectin (L/A) ratio according to dementia and abdominal obesity (AbdOb). Data are presented as geometric means with interquartile ranges. Values in parentheses are adjusted on age, sex, anti-diabetic, anti-hypertensive and lipid-lowering treatment (ANCOVA)

The comparison of investigated adipokines levels between subjects with and without abdominal obesity revealed significant differences concerning adiponectin and leptin but not resistin levels, both in all-cause dementia and controls. Adiponectin levels were significantly lower, while leptin levels were significantly higher in subjects with abdominal obesity (Fig. 1).

Moreover, in all-cause dementia obese patients compared to patients without abdominal obesity significantly higher levels of pro-inflammatory indices (IL-6 and hsCRP) and lower anti-inflammatory parameters (HDL-C) were observed. Higher levels of BMI and parameters associated with glucose metabolism (the post-load glucose, insulin and HOMA-IR) were also found in abdominal obesity. In the control group the relationships were less pronounced (data not shown).

To examine the possible influence of the abdominal obesity on the differences in adipokines levels between all patients with dementia and controls we stratified study subjects into subgroups with and without abdominal obesity (Fig. 1). This allowed to demonstrate significant differences in the concentrations of adiponectin between the whole dementia group and controls (i.e. the higher levels of adiponectin in dementia) only in subjects without abdominal obesity. The significantly lower levels of L/A ratio in dementia as compared with controls was observed also only in subjects without abdominal obesity. Interestingly, statistically significant differences in concentrations of leptin between all-cause dementia and controls appeared only after the stratification by abdominal obesity. The statistically significant lower level of leptin in all-cause dementia in comparison with controls was found only in those without abdominal obesity [4.32 ng/mL (2.33–8.09) vs. 5.81 ng/mL (2.98–11.09), p = 0.042]. There was no significant influence of abdominal obesity on resistin levels in the whole dementia group. The higher levels of resistin in dementia were stated both in subjects with and without abdominal obesity.

The influence of both abdominal obesity and dementia status on serum adipokines levels was confirmed by a Two-way analysis of variance (Fig. 1). The analysis showed significant main effects of both the presence of dementia and the abdominal obesity on adiponectin levels. The level of leptin depended only on the presence of abdominal obesity, but not dementia status. Moreover, in the case of leptin levels a significant interaction between dementia status and abdominal obesity was shown (p = 0.018) indicating the opposite direction in differences of leptin levels in all-cause dementia, dependent on abdominal obesity presence i.e. tendency to higher leptin levels in demented patients with obesity and significantly decreased level in demented patients without abdominal obesity (as compared to appropriate control groups with and without abdominal obesity). Similarly to leptin the L/A ratio depended only on the presence of abdominal obesity, but not dementia status and the significant interaction between dementia status and abdominal obesity was shown (p = 0.007). The level of resistin depended only on dementia status, and not on the presence of abdominal obesity.

The similar influence of the presence of MetS on investigated adipokines levels in dementia was observed (data not shown).

### Correlations of adipokines with glucose metabolism parameters and inflammatory indices in patients with dementia and controls

The correlation coefficients of adiponectin, leptin and resistin with selected metabolic parameters and inflammatory indices in the entire dementia group and controls are shown in Table 5.

**Table 5:** Univariate correlation analysis

	Dementia (n = 205)			Controls (n = 107)		
	Adiponectin	Leptin	Resistin	Adiponectin	Leptin	Resistin
Age						
R	0.132	0.034	0.300	0.025	−0.064	0.294
p value	0.059	0.633	0.00001	0.797	0.516	0.002
MMSE						
R	−0.119	0.063	0.007	–	–	–
p value	0.089	0.372	0.926			
BMI						
R	−0.519	0.644	0.014	−0.324	0.529	0.034
p value	<0.000001	<0.0000001	0.837	0.0007	<0.0000001	0.729
Glucose 0 h						
R	−0.234	0.184	−0.003	−0.293	0.264	0.075
p value	0.0007	0.008	0.971	0.002	0.006	0.442
Insulin						
R	−0.360	0.402	−0.057	−0.409	0.228	0.084
p value	<0.000001	<0.0000001	0.415	0.00001	0.018	0.390
HOMA-IR						
R	−0.402	0.415	−0.046	−0.457	0.278	0.099
p value	<0.000001	<0.000001	0.514	0.000001	0.004	0.309
IL-6						
R	−0.187	0.153	0.340	−0.224	0.023	0.260
p value	0.007	0.029	0.0000001	0.020	0.812	0.007
hsCRP						
R	−0.170	0.314	0.234	−0.096	0.205	0.048
p value	0.015	0.00004	0.0007	0.324	0.034	0.626
25-OH Vitamin D						
R	0.082	−0.085	−0.132	−0.027	−0.099	0.018
p value	0.244	0.227	0.060	0.780	0.309	0.853
PON1						
R	−0.024	0.042	−0.236	0.099	0.107	−0.166
p value	0.727	0.545	0.0006	0.310	0.272	0.088
HDL-C						
R	0.499	−0.263	−0.301	0.311	0.025	0.094
p value	<0.0000001	0.0001	0.00001	0.001	0.801	0.334
Chitotriosidase						
R	−0.035	0.077	0.165	0.125	0.125	−0.093
p value	0.617	0.274	0.018	0.201	0.199	0.341

Spearman’s correlation coefficients (R) of adiponectin, leptin and resistin level with selected metabolic parameters and inflammation indices in dementia and controls

*BMI* body mass index, *CRP* C-reactive protein, *HDL-C* high density lipoprotein cholesterol, *HOMA-IR* homeostatic model assessment index, *IL-6* interleukin 6, *MMSE* mini mental state examination, *PON1* paraoxonase 1

A significant negative correlation of adiponectin levels with BMI and glucose metabolism parameters (fasting glucose, insulin and HOMA-IR index) and with pro-inflammatory indices (IL-6 and hsCRP) and the positive correlation with HDL-cholesterol level (considered as negative indicator of inflammation) was found in the whole dementia group. Borderline negative correlation of resistin with MMSE score (indicating the severity of dementia) was observed. In controls, similar results were obtained, with exception of hsCRP.

In the case of leptin, as compared to adiponectin, the opposite direction of correlations with the same parameters were stated in all-cause dementia i.e. positive correlation with BMI, fasting glucose, insulin, HOMA-IR, IL-6 and hsCRP and positive correlation with HDL-C. In controls similar results were obtained with exception of IL-6 and HDL-C. As expected, the significant negative correlation between leptin and adiponectin was stated in dementia (R = −0.327, p = 0.000002; data not shown).

Highly significant positive correlation between the level of resistin and age and indices of inflammation (IL-6 and hsCRP, chitotriosidase activity) and a negative correlation with HDL-cholesterol and PON1 activity was stated in patients with all-cause dementia. Borderline negative correlation was observed with 25-OH vitamin D serum levels. In the control group the significant positive correlation of resistin with age and IL-6 was demonstrated. Moreover, we did not observe any correlation between resistin and adiponectin and leptin, both in whole dementia and control group (data not shown). 

Multivariate regression analysis (Table 6) performed in the whole group of investigated individuals demonstrated significant positive associations between adiponectin and HDL-C, age, AD and MD status and negative associations with HOMA-IR, male sex, anti-diabetic therapy and BMI. It was shown that the variables included in the model explained 46.2% of the variance of serum adiponectin levels. In the case of leptin significant positive associations with BMI, HOMA-IR and hsCRP and negative association with male sex were found. The variables included in the model explained 63.4% of the variance of serum leptin levels. The analysis showed the significant positive associations between resistin and serum creatinine, age, IL-6 and VaD and MD status and negative associations with male sex. It was shown that the variables included in the model explained 21.9% of the variance of serum resistin levels.

**Table 6:** Factors affecting adiponectin, leptin, and resistin levels in whole investigated group (n = 425) based on multivariate stepwise regression analysis

Variables	Adiponectin^a^			Leptin^a^			Resistin^a^		
	β	ΔR^2^	p	β	ΔR^2^	p	β	ΔR^2^	p
HDL-C^a^	0.282	0.232	<0.000001						
HOMA-IR^a^	−0.213	0.087	<0.000001	0.207	0.035	<0.000001	–	–	–
Age	0.210	0.053	<0.000001				0.156	0.054	0.00001
Anti-diabetic therapy	−0.197	0.044	<0.000001						
Sex	−0.183	0.026	0.00002	−0.452	0.182	<0.00001	−0.149	0.010	0.023
BMI	−0.115	0.012	0.003	0.569	0.414	<0.00001	–	–	–
AD	0.111	0.007	0.022						
MD	0.140	0.009	0.008				0.101	0.006	0.037
VaD							0.141	0.012	0.015
IL-6^a^				–	–	–	0.123	0.022	0.001
hsCRP^a^	–	–	–	0.084	0.006	0.007	–	–	–
Creatinine							0.353	0.120	<0.00001
	R^2^ adj = 0.462, p < 0.00001			R^2^ adj = 0.634, p < 0.00001			R^2^ adj = 0.219 p < 0.00001		

Sex (female = 0; male = 1).

Adiponectin: variables included in the model: age, sex, BMI, HDL cholesterol (HDL-C), HOMA-IR, hsIL-6, cognitive status (AD, MD, VaD, MCI) and anti-diabetic, anti-hypertensive and lipid-lowering therapy.

Leptin: variables included in the model: age, sex, BMI, HDL cholesterol (HDL-C), HOMA-IR, hsCRP, cognitive status (AD, MD, VaD, MCI) and anti-diabetic, anti-hypertensive and lipid-lowering therapy and cognitive status (AD, MD, VaD, MCI) and anti-diabetic, antihypertensive and lipid-lowering therapy.

Resistin: variables included in the model: age, sex, HDL cholesterol (HDL-C), IL-6, 25(OH)vitamin D, creatinine, serum chitotriosidase, current smoking, cognitive status (AD, MD, VaD, MCI) and anti-diabetic, anti-hypertensive and lipid-lowering therapy

*β* the standard regression coefficient, *R* ^*2*^ *adj* the multiple coefficient of determination (adjusted), *AD* Alzheimer’s disease, *BMI* body mass index, *CRP* C-reactive protein, *HDL-C* high density lipoprotein cholesterol, *HOMA-IR* homeostatic model assessment index, *IL-6* interleukin 6, *MD* mixed dementia, *MMSE* mini mental state examination, *VaD* vascular dementia

^a^Logarithmically transformed in statistical analysis

## Discussion

Risk factors for all-cause dementia, AD and dementia with vascular changes in the brain exhibit overlap with those for cardiovascular disease. One of the several important risk factors is metabolic syndrome and abdominal obesity associated with deregulation of adipokines-substances secreted by adipocytes and immune cells (Frisardi et al. 2010; Parimisetty et al. 2016).

The main finding of the present study was to establish the association between dementia and adipokines.

Significantly increased adiponectin levels were observed in all-cause dementia, particularly in dementia of neurodegenerative origin (AD and MD) as compared to controls. This observation was confirmed by logistic regression analysis and the multivariate regression analysis. The same results were obtained in previous studies showing an association of higher circulating (or cerebrospinal fluid) adiponectin level with increased risk of AD (Une et al. 2011; van Himbergen et al. 2012; Khemka et al. 2014). On the contrary, other authors Teixeira et al. (2013) observed that decreased adiponectin serum concentrations are associated with MCI and AD, but do not predict cognitive decline in elderly individuals. Recently a study of the high molecular-weight adiponectin (HMW) level and incident dementia in patients with vascular risk factors showed little association with future dementia (Kitagawa et al. 2016).

Interestingly, the increased adiponectin observed in our study in all-cause dementia was significant only in individuals without abdominal obesity or MetS. In a recently published review, Ishii and Iadecola (2016) speculate that the association of increased plasma adiponectin levels with dementia found in some studies, may be surprising for the first moment, since low adiponectin levels are often associated with obesity-associated disorders and adiponectin is generally considered to have protective properties. However, these studies were conducted predominately in patients with AD, where weight loss is characteristic feature. This condition could be associated with higher circulating adiponectin levels. High circulating adiponectin levels may lead to subsequent resistance to adiponectin in a similar fashion to insulin resistance and leptin resistance (Ishii and Iadecola 2016).

In the present study we found only a borderline significant negative correlation between adiponectin and severity of dementia measured with MMSE, which is in agreement with previous studies of Bigalke et al. (2011), who observed an inverse trend to a lower MMSE score with increased plasma levels of adiponectin. In contrast, the Khemka et al. (2014) study demonstrated a positive correlation of adiponectin with MMSE.

In addition, both in dementia and non-demented elderly controls, we observed a high negative correlation between adiponectin and metabolic parameters i.e. BMI, fasting glucose, insulin and HOMA-IR index. Similar observations were previously stated in non-demented subjects (Zoico et al. 2004; Hung et al. 2008). A significantly lower level of adiponectin was associated with the presence of abdominal obesity in all our investigated groups, which was confirmed by a Two-way ANOVA analysis.

We also demonstrated negative correlation of adiponectin with pro-inflammatory indices (IL-6 and hsCRP) and positive correlation with HDL cholesterol, a negative indicator of inflammation. These findings are in agreement with previous observations that adiponectin as an anti-inflammatory factor decreases the expression of pro-inflammatory cytokines and increases the expression of anti-inflammatory molecules (Tilg and Moschen 2006; Hung et al. 2008).

The results of the current study did not show significant differences in the levels of leptin between all-cause dementia, different types of dementia, MCI and the control group of elderly subjects. This observation was confirmed by logistic regression analysis and the multivariate regression analysis. These findings are in agreement with results of some previous studies performed in clinical AD (Theodoropoulou et al. 2012; Warren et al. 2012; Teunissen et al. 2015), but different to results of other authors. The results of two large population based prospective studies provided the evidence for a lower incidence of AD in elderly individuals associated with higher leptin levels (Holden et al. 2009; Lieb et al. 2009). Several case–control studies showed that AD patients had significantly decreased plasma levels of leptin compared with healthy controls (Bigalke et al. 2011; Baranowska-Bik et al. 2015).

The interesting observation that higher leptin level was associated with better cognitive function only in subjects without central obesity was stated by Hazzouri et al. (2013), suggesting that obesity may interfere with the neuroprotective effect of leptin on the brain, possibly by leptin resistance. In another small case–control study Power et al. (2001) showed that below-appropriate-weight patients with dementia, both AD and VaD, had decreased leptin levels in comparison with appropriate-weight controls. Similarly, in our study we observed significantly lower serum leptin level in whole dementia group in comparison with control subjects only in individuals without abdominal obesity. It was confirmed by high positive correlation of leptin and BMI in all investigated groups.

In our present study we did not reveal any significant correlation between leptin and severity of dementia measured with MMSE, which is in agreement with previous studies of Baranowska-Bik et al. (2015) and Teunissen et al. (2015) performed in patients with AD. In contrast, in other studies the positive (Bigalke et al. 2011) or negative (Khemka et al. 2014) correlation of leptin levels with MMSE was found.

Moreover, both in all dementia patients and non-demented elderly controls, we confirmed a high positive correlation between leptin and selected metabolic parameters i.e. BMI, fasting glucose, insulin and HOMA-IR index previously stated in elderly subjects (Zoico et al. 2004). Significantly higher levels of leptin were associated with the presence of abdominal obesity in all investigated groups, which was confirmed by a Two-way ANOVA analysis.

We also demonstrated a positive correlation of leptin with some pro-inflammatory indices IL-6 and hsCRP and negative correlation with HDL cholesterol considered as a negative indicator of inflammation. These findings are in agreement with previous studies showing leptin to be a pro-inflammatory cytokine (Tilg and Moschen 2006).

Leptin has been demonstrated to have a neuroprotective role against AD pathology and dementia. One has to mention that leptin was considered to be a potential cognitive enhancer (Harvey et al. 2005). It was shown in experimental studies that leptin reduced both brain Aβ load (Fewlass et al. 2004) and hyperphosphorylated tau in neuronal cells (Greco et al. 2008), thus diminishing two important elements of Alzheimer’s disease. Moreover, the results from animal studies demonstrated a role of leptin in memory decline and AD pathology, e.g., showing that treatment of transgenic mouse model of Alzheimer’s disease with leptin led to a reduction of Aβ levels and memory improvement (Greco et al. 2010). These observations made leptin a potential therapeutic agent (Tezapsidis et al. 2009). It was shown that obese people are likely to develop leptin resistance, the condition which may attenuate the beneficial action of leptin on the brain and may be responsible for the lack of protective effect of leptin on dementia and Alzheimer’s disease among obese people. In non-obese people, leptin may exert its neuroprotective effect (Ishii and Iadecola 2016).

The most interesting observation in our study is the presence of elevated levels of resistin in all patients with dementia, particularly in dementia with vascular changes in the brain (VaD and MD) in comparison with non-demented controls. This observation was confirmed by the multivariate regression analysis. In logistic regression analysis crude ORs showed significant association of increased levels of resistin with higher risk of all-cause dementia, MD and VaD, however these associations were no longer statistically significant after controlling for the other risk factors.

The relation of circulating resistin with dementia was studied by other authors but the results of studies that have since been published are inconclusive. A recent population-based longitudinal study performed in 933 subjects over 50 years with moderate to high vascular risk suggested that resistin was not related to cognitive performance measured in different cognitive domains tests (Mirabell et al. 2013). In another small clinical study performed in 38 AD patients and 32 control subjects increased serum resistin levels were observed to be associated with AD. The authors claimed that resistin is a regulatory marker of inflammation and considered resistin being a predictor of Alzheimer’s disease caused by activation of the immune system. Based on high sensitivity, specificity and predictive values the authors tried to apply plasma resistin determination as a diagnostic marker for AD (Kizilarslanoglu et al. 2015).

In the present study, in opposition to leptin and adiponectin (both in dementia and non-demented elderly controls) we did not observe any correlation between resistin and metabolic parameters i.e. BMI, fasting glucose, insulin and HOMA-IR index. These findings are in agreement with the results of several previous studies performed in various populations (Lee et al. 2003; Bo et al. 2005; reviewed in Schwartz and Lazar 2011). We also did not demonstrate any association of resistin levels with abdominal obesity, neither in all-cause dementia nor in controls, demonstrated after stratification of the data by abdominal obesity, which was confirmed by a Two-way ANOVA analysis.

In the present study, we did show a positive correlation of resistin with inflammatory indicators (interleukin-6 and CRP) and a negative correlation with anti-inflammatory markers (HDL-C and PON1 activity) in all dementia patients. In non-demented controls, only positive correlation with IL-6 reached statistical significance. These results may suggest that resistin could be a remarkable inflammatory marker in dementia. The exact role of resistin in inflammation was not explained and it is still unclear whether it is a marker of inflammation or a pathogenic factor (Karbowska et al. 2009). Some authors stated that it was secreted as a marker of chronic inflammation (Bo et al. 2005; Olefsky and Glass 2010) and on the other hand it was considered to be a factor causing enhanced production and secretion of pro-inflammatory cytokines (Silswal et al. 2005).

Another interesting finding in our study is the significant positive correlation of resistin level with serum chitotriosidase activity stated in all patients with dementia, particularly in dementia with vascular changes. Chitotriosidase (CHIT1) is an enzyme involved in the degradation of chitin and chitin-like substrates. CHIT1 activity is increased in several lineages of activated macrophages which are involved in both physiological and pathologic processes. CHIT1 expression is altered in a number of non-infectious inflammatory diseases. Being a nonspecific marker of macrophage activation, recently the enzyme is considered as a novel marker of inflammation (Kanneganti et al. 2012). The increased circulating CHIT1 activity is a biomarker of lysosomal storage diseases such as Gaucher’s disease and is used to monitor progression or the efficacy of treatment with enzyme replacement therapy (Hollak et al. 1994). Elevated serum CHIT1 activities were also reported in atherosclerosis (both coronary and cerebrovascular), and Alzheimer’s disease (Elmonem et al. 2016). The positive correlation between resistin and CHIT1 stated in our study confirm the association of elevated resistin levels with activation of macrophages and inflammation.

Because a tremendous increase of dementia cases and the lack of its effective treatment there is an urgent need to recognize all the factors accelerating or inhibiting its development. In the present study several important factors present in blood serum were analyzed: the hormones produced by adipose tissue (adiponectin, leptin and resistin), pro-inflammatory and anti-inflammatory markers and glucose metabolism parameters in various types of dementia, mild cognitive impairment and controls to identify a characteristic profile for dementia and its type. The role of adiponectin in dementia with neurodegenerative symptoms was confirmed. A very important observation concerns resistin which was stated to be a significant marker of inflammation in dementia especially in dementia of vascular mechanism. Identification of this novel biomarker may be important in preventing dementia.

Our study has some limitations that are worth noting.

First of all in our sample, the all-cause dementia group patients (particularly the MD) were significantly older than non-demented controls and all groups with dementia were also significantly less educated. As age and education are known to be independent predictive factors for cognitive decline, they may influence some of the other comparisons. For example, our younger non-demented controls might be persons who have not yet developed dementia. Adjustment for age and education level was taken into consideration in ANCOVA and Logistic regressions analyses.

Second, it is known that exercise training could influence the lipid, glucose metabolism and inflammatory markers levels. But the precise information about the physical activity of the participants of our study is lacking and is based only on the neuropsychological anamnesis which showed that over 90% of participants were retired and in most of them physical activity is limited to daily activities.

Third, the sample size in the case of leptin analyses seems too small to reach statistical significance of the results. The calculated power of the ANOVA test used to compare adiponectin, leptin and resistin levels between the investigated groups was 76.2, 18.5% and 94.9%, respectively. The calculated power of the test used in analysis for leptin is very low. The reason could be the high within-subject variability of leptin levels observed in all investigated groups. The exceedingly high variability of leptin levels could be the fact that leptin levels may be subject to diurnal variation. However, this does not bias our results since leptin was measured at the same time (fasting in the morning) in all our study participants. Assuming that the power of the test should be at least 80%, calculated size of the groups in the case of leptin ANOVA analysis should exceed 229 individuals in each group. We recommend the design of larger case–control studies to further address this issue.

Fourth, we did not investigate the serum level of specific adiponectin isoforms. These isoforms may have distinct biologic effects, for example, the high molecular weight isoform has higher biological activity than the low molecular weight isoform (Swarbrick and Havel 2008). Therefore, additional studies are necessary to evaluate the role of specific adiponectin isoforms in the development of dementia.

## Conclusions

1. Dementia of neurodegenerative origin (Alzheimer’s disease and mixed dementia) is characterized by elevated levels of adiponectin, whereas dementia with vascular changes in the brain (vascular and mixed dementia) is characterized by an increase of resistin.
2. Positive correlation of resistin with inflammation indicators and negative correlation with the compounds with anti-inflammatory properties may suggest the potential pro-inflammatory role of resistin in the development of dementia, especially in dementia of vascular mechanism.
3. The opposite direction of correlations of adiponectin and leptin with inflammatory markers in all-cause dementia confirmed previous findings showing adiponectin to have anti-inflammatory and leptin to have pro-inflammatory properties.
4. Significant associations of abdominal obesity and glucose metabolism parameters with adiponectin and leptin but not resistin levels were stated.
